# A long-term reconstructed TROPOMI solar-induced fluorescence dataset using machine learning algorithms

**DOI:** 10.1038/s41597-022-01520-1

**Published:** 2022-07-20

**Authors:** Xingan Chen, Yuefei Huang, Chong Nie, Shuo Zhang, Guangqian Wang, Shiliu Chen, Zhichao Chen

**Affiliations:** 1 State Key Laboratory of Hydroscience and Engineering, Department of Hydraulic Engineering, Tsinghua University, Beijing, 100084 China; 2 The Key Laboratory of Ecological Protection and High Quality Development in the Upper Yellow River, Qinghai Province, China; 3 State Key Laboratory of Plateau Ecology and Agriculture, Qinghai University, Xining, Qinghai 810016 China; 4 Chinese Research Academy of Environmental Sciences, Beijing, 100012 China; 5 National Joint Research Center for Yangtze River Conservation, Beijing, 100012 China

**Keywords:** Ecosystem ecology, Phenology

## Abstract

Photosynthesis is a key process linking carbon and water cycles, and satellite-retrieved solar-induced chlorophyll fluorescence (SIF) can be a valuable proxy for photosynthesis. The TROPOspheric Monitoring Instrument (TROPOMI) on the Copernicus Sentinel-5P mission enables significant improvements in providing high spatial and temporal resolution SIF observations, but the short temporal coverage of the data records has limited its applications in long-term studies. This study uses machine learning to reconstruct TROPOMI SIF (RTSIF) over the 2001–2020 period in clear-sky conditions with high spatio-temporal resolutions (0.05° 8-day). Our machine learning model achieves high accuracies on the training and testing datasets (R^2^ = 0.907, regression slope = 1.001). The RTSIF dataset is validated against TROPOMI SIF and tower-based SIF, and compared with other satellite-derived SIF (GOME-2 SIF and OCO-2 SIF). Comparing RTSIF with Gross Primary Production (GPP) illustrates the potential of RTSIF for estimating gross carbon fluxes. We anticipate that this new dataset will be valuable in assessing long-term terrestrial photosynthesis and constraining the global carbon budget and associated water fluxes.

## Abstract (summary)

**Table:** 

Measurement(s)	Solar-Induced Fluorescence
Technology Type(s)	machine learning algorithms

## Background & Summary

Accurate quantification of gross primary production (GPP) through photosynthesis is essential for studies of ecosystem function, carbon cycle, human welfare, and net-zero carbon emission^1–4^. Various methods have been developed to estimate GPP at the global scale, which can be divided into three main categories: enzyme kinetic (process-based) models^5–7^, light use efficiency (LUE) models^8–12^, and data-driven approaches^13–17^. While a wide range of global GPP estimates is available, the significant discrepancies in GPP estimates generated by different methods remain one of the most uncertain aspects in quantifying the global carbon cycle^18–22^. Over the past decade, advances in global remote sensing of solar-induced chlorophyll fluorescence (SIF) have made it possible to inform on vegetation photosynthetic activity at a global scale^23–30^, providing new opportunities for accurate GPP estimates.

SIF is a small fraction of re-emitted light accompanying the absorption of photosynthetically active radiation (PAR) by excited chlorophyll-a molecules in the spectral range from 650 to 800 nm^31^. The first approved global mission designed explicitly for SIF measurement of terrestrial vegetation, the FLuorescence EXplorer (FLEX), was selected as the eighth Earth Explorer mission of the European Space Agency and will be launched in 2025^32^. The global SIF datasets currently used are estimated from atmospheric sensors because they have the required spectral resolution and signal-to-noise ratio (details of the sensors are given in Table 1). However, the existing SIF records have long been limited by their low spatial resolution and sparseness in data acquisition. For instance, the Global Ozone Monitoring Experiment-2 (GOME-2)^24^ and the SCanning Imaging Absorption SpectroMeter for Atmospheric CHartographY (SCIAMACHY)^26^ provide spatially continuous coverage of SIF but with large footprint size (hence low spatial resolution, Table 1). Conversely, the Greenhouse Gases Observing Satellite (GOSAT)^23^ and the Orbiting Carbon Observatory-2 (OCO-2)^25^ offer much smaller footprint size, but with sparse and thus spatially discontinuous measurements.

**Table 1:** Space-borne instruments currently in orbit enabling SIF estimation.

Sensor	GOSAT	GOME-2	SCIAMACHY	OCO-2	TanSat	TROPOMI	FLEX
Launch time	2009/6	2007/1	2002/3	2014/7	2016/12	2017/10	2025
Overpass time	13:30	9:30	9:30	13:15	13:00	13:30	10:00
Spatial coverage	sparse	continuous	continuous	sparse	sparse	continuous	continuous
Footprint size	10 km	40 × 80 km	30 × 240 km	1.5 × 2.25 km	2 × 2 km	3.5 × 5.5 km	300 m
Temporal resolution	3 days	1.5 days	6 days	16 days	16 days	1 day	27days

The above dilemma is partially addressed by the TROPOspheric Monitoring Instrument (TROPOMI) on the Copernicus Sentinel-5P mission thanks to the significantly increased spatiotemporal resolution and data coverage^27^. TROPOMI has almost global coverage (except for small gaps between footprints) and high spatial resolution (3.5 km × 5.5 km at nadir)^33^. Compared with the earlier missions, TROPOMI has a daily revisit time to provide a significant increase in the number of clear-sky measurements. However, Sentinel-5P was launched in October 2017, and the TROPOMI SIF data are only available since April 2018, limiting its use for long-term applications.

This study uses machine learning algorithms to reconstruct TROPOMI SIF (RTSIF) for a longer period to alleviate the issue above. RTSIF is generated based on the Caltech TROPOMI SIF data^27^, the nadir bidirectional reflectance distribution adjusted reflectance (NBAR)^34^, land surface temperature (LST)^35^, and land cover data^36^ from the Moderate Resolution Imaging Spectroradiometer (MODIS), the PAR data^37^ from the Earth’s Radiant Energy System (CERES), and the vegetation type data^38^ from the International Satellite Land Surface Climatology Project, Initiative II (ISLSCP II). This dataset extends the time coverage of the TROPOMI SIF data and provides a long-term, high-resolution, and global SIF record. RTSIF is in good agreement with TROPOMI SIF and has been evaluated against the GOME-2 and OCO-2 SIF. We further demonstrate the consistency between RTSIF and tower measured SIF and GPP. The proposed dataset provides a new dataset for SIF evaluation and could benefit related ecosystem, carbon cycle, and net-zero carbon emission studies.

## Methods

### Framework overview

**Fig. 1:**
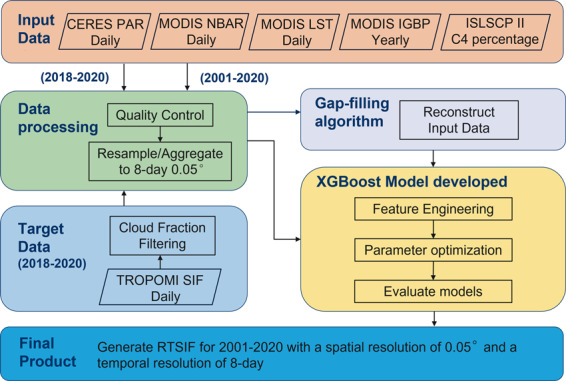
The workflow to generate RTSIF.

Figure 1 illustrates the overall framework used to generate RTSIF. Based on the LUE concept, SIF can be expressed as follows according to Zhang *et al*.^39^ and Zhang *et al*.^40^:

1 $${\rm{SIF}}={\rm{PAR}}\times {{\rm{fPAR}}}_{{\rm{chl}}}\times {\rm{FE}}$$

where fPAR_chl_ is the fraction of PAR absorbed by chlorophyll (${{\rm{APAR}}}_{chl}$) and FE is the fluorescence efficiency. Since SIF originates from the solar energy absorbed by chlorophyll-a molecules^41^, it is highly correlated with ${{\rm{APAR}}}_{chl}$, the product of ${{\rm{fPAR}}}_{chl}$ and PAR^42–44^. Previous studies have shown that ${{\rm{fPAR}}}_{chl}$ can be estimated from surface reflectance using radiative transfer models^45^, and thus PAR and surface reflectance have been widely used to reconstruct SIF^46–51^. Previous studies have also shown that the high correlation between SIF and APAR_chl_ is limited to unstressed conditions^52^, while drought and other environmental stresses can affect FE. LST can be used as a proxy of thermal stress in predictive models of SIF^53–56^. In this study, we further consider that including biome type may improve the prediction accuracy of the SIF model given the plant structural and physiological differences in different biomes and different photosynthetic pathways in C3 and C4 plants. We finally selected surface reflectance, PAR, LST, land cover, and C3/C4 fraction as input variables for the RTSIF modeling.

### Data Sets

We used multiple datasets as input to generate RTSIF. All the datasets used are summarized in Table 2 and described in detail as follows.

**Table 2:** Datasets used in developing the machine learning model for RTSIF and their characteristics.

Data source	Dataset	Derived variables	Spatial resolution	Temporal resolution	Available at
TROPOMI	TROPOMI SIF	SIF	ungridded	Daily	ftp://fluo.gps.caltech.edu/data/tropomi/
MODIS	MOD11C1	LST	0.05° × 0.05°	Daily	10.5067/MODIS/MOD11C1.006^85^
	MCD12C1	Land cover	0.05° × 0.05°	Yearly	10.5067/MODIS/MCD12C1.006^86^
	MCD43C4	NBAR	0.05° × 0.05°	Daily	10.5067/MODIS/MCD43C4.006^87^
CERES	SYNI	PAR	1° × 1°	Daily	10.5067/Terra+Aqua/CERES/SYN1degDay_L3.004A^88^
ISLSCP II	C4 vegetation percentage map	C4 percentage	1° × 1°	invariant	10.3334/ORNLDAAC/932^82^

The Caltech TROPOMI SIF data between March 2018 and December 2020 were used for model training and evaluation. We followed the filtering scheme in the original reference^27^ to retain daily average clear-sky SIF data with cloud fractions less than 0.1, and excluded the data with a sun zenith angle (SZA) greater than 60° and a view zenith angle (VZA) greater than 70°. The ungridded data through the filtering scheme were aggregated to 0.05° grids at an 8-day resolution, the grid size of which was close to the footprint size of the TROPOMI SIF data. Averaging the multiple observations reduces the uncertainty in the original SIF retrievals by $\sqrt{n}$ (n is the effective number of observations in the grid cell)^25^. For each 0.05° grid, only the SIF footprint covering the center of the grid was recorded as valid retrievals, and the SIF values were calculated only when more than four valid retrievals were included. We used the SIF values at 740 nm from the 743–758 nm retrieval window, which is optimal for high retrieval precision and low sensitivity to clouds^33^.

Ancillary input data including the MODIS land products, the CERES products, and the ISLSCP II products were used to generate RTSIF. The MODIS products included LST (MOD11C1^35^), land cover (MCD12C1^36^), and seven bands for nadir bidirectional reflectance distribution adjusted reflectance (NBAR; MCD43C4^34^). To reduce the uncertainty in the SIF modeling, only high-quality MOD11C1 (QA < 2) and MCD43C4 (QA < 2) data were used and aggregated to an 8-day average. Gap-filling and smoothing algorithms were used to reconstruct the 8-day MOD11C1 and MCD43C4 data^57^ and replace the poor observations caused by bad atmospheric conditions. We used an updated land cover map (MCD12C1) for each year. PAR data (SYNI PAR^37^) from the CERES products were used, aggregated to 8-day, and interpolated to 0.05° using bilinear interpolation. The ISLSCP II C4 vegetation map was used for natural C4 vegetation distribution^38^, assuming that all the vegetation types within each 1° grid cell shared the same C3/C4 ratio.

### Data-Driven approach

Extreme Gradient Boosting (XGBoost) is an enhanced version of the machine learning algorithm named Gradient Boosted Decision Tree (GBDT)^58^. It constructs enhanced trees that can handle complex nonlinear relationships^59,60^. As a boosting algorithm, XGBoost consists of multiple decision trees, each of which is trained with the residual error of the predicted result from the previous decision tree, and finally iterates the results of all the decision trees before producing the final result. Compared with other traditional GBDT algorithms that only use first-order derivatives, XGBoost performs a second-order Taylor expansion on the loss function between computed results and actual observations to accelerate the convergence of the model during training and provide higher efficiency in finding the optimal solution. In addition, XGBoost has a regularization term to control the complexity of the model, which can effectively avoid overfitting. The TROPOMI SIF and the input variables constitute a dataset containing a large number of data samples (about 36 million). The current machine learning algorithms have difficulties in processing large datasets using existing packages^61^, while XGBoost employs software and hardware optimization techniques to make it possible to process tens of millions of training data. In this study, XGBoost was implemented using the Python library XGBoost (https://github.com/dmlc/xgboost). Before training, each variable was standardized by its mean and deviation. We split the data into the training group (80%) and the testing group (20%). Many hyperparameters in XGBoost affect the model performance, and a grid search was performed for the hyperparameters with 10-fold cross-validation to find the best combination of the parameters based on the Root Mean Square Error (RMSE) metric^62^. The optimized hyperparameters are compiled in Supplementary Table S1.

## Data Records

Our long-term global SIF dataset, RTSIF, is available at 10.6084/m9.figshare.19336346.v2^63^. The data record contains global RTSIF data from January 2001 to December 2020 at a 0.05°/8-day resolution. There are 46 GeoTiff files per year, one for each 8-day period. The unit is mWm^−2^nm^−1^sr^−1^. The file name RTSIF_ < YYYY > - < MM > - < DD > .tif provides information on the year, month, and start date of the 8-day period. Considering that deserts and glaciers have no vegetation, those pixels are flagged.

## Technical Validation

### Model validation

**Fig. 2:**
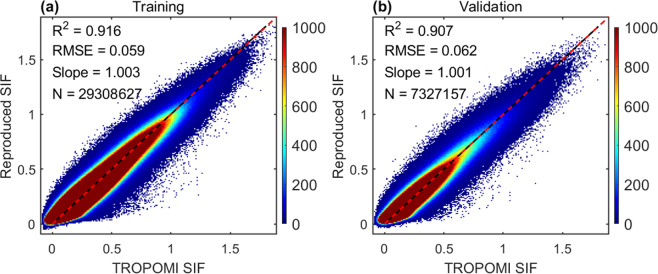
Performance of the XGBoost model in reproducing TROPOMI SIF over the training and testing data. The shading color represents the density of the scatterplot. Black lines represent the regression slope, and the red dotted lines represent the 1:1 line. The regression is forced to pass the origin. All values are in the unit of mWm^−2^nm^−1^sr^−1^.

We tested the performance of the XGBoost model with the optimal hyperparameters. The model reproduces the TROPOMI SIF with a determination coefficient R^2^ of 0.916, a RMSE of 0.059 mWm^−2^nm^−1^sr^−1^ during training, and an R^2^ of 0.907, and an RMSE of 0.062 mWm^−2^nm^−1^sr^−1^ during testing (Fig. 2), suggesting that our optimized XGBoost model is not overfitting. The slope of the fit between the reproduced and observed SIF values is close to 1, indicating that there is no systematic discrepancy. We also investigated the performance of the model for each land cover type defined in the MCD12C1 dataset. For most land cover types, the reproduced and observed TROPOMI SIF values have R^2^ values over 0.8 (Table. S2).

**Fig. 3:**
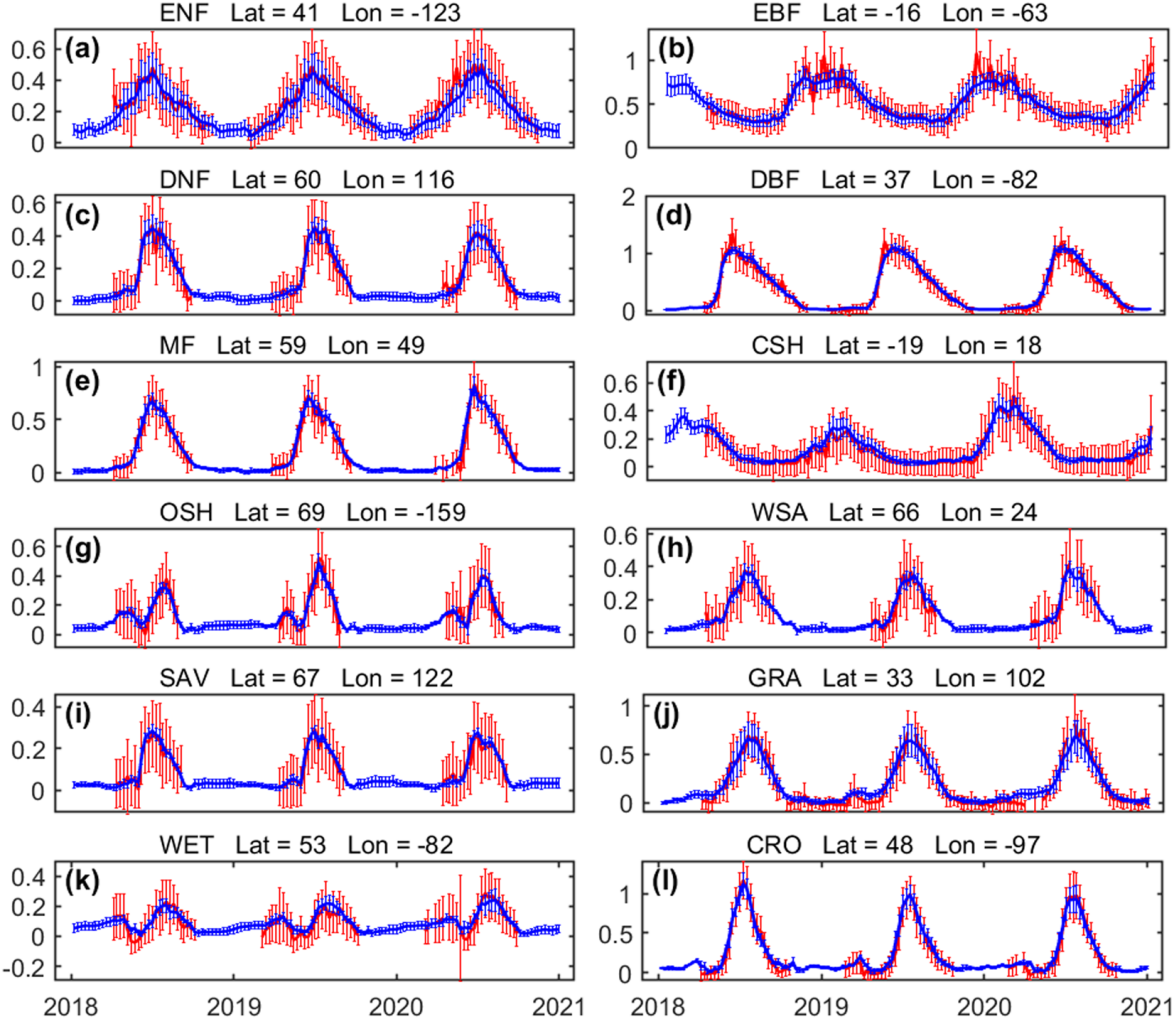
Time series of RTSIF and TROPOMI SIF for selected 1° grid cells. All the samples from the training data and the testing data were used. The red line represents TROPOMI SIF, and the blue line represents RTSIF. The error bars represent the standard deviation of the TROPOMI SIF footprint and RTSIF used to generate 1° grid. The MODIS MOD12C1 land cover dataset was used to select these example grid cells. All the values are in the unit of mWm^−2^nm^−1^sr^−1^.

We compared RTSIF and TROPOMI SIF for 1° selected grid cells representative of the 12 vegetated biomes (locations shown in Fig. S1b). RTSIF can accurately capture seasonal and interannual variations in TROPOMI SIF for most biome types. The standard deviation in the RTSIF data is typically smaller than that in the originally retrieved TROPOMI SIF, indicating reduced noise in the RTSIF dataset. RTSIF also fills the gaps where no TROPOMI SIF data are available (Fig 3).

**Fig. 4:**
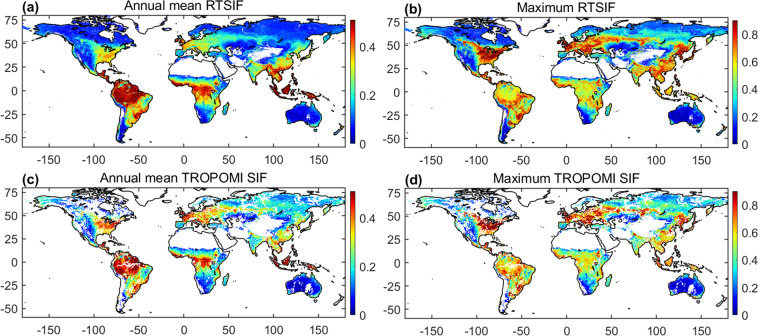
Spatial pattern of average and maximum (90^th^ percentile) daily values for RTSIF (**a** and **b**) and TROPOMI SIF (**c** and **d**) in 2019. All the values are in units of mWm^−2^nm^−1^sr^−1^.

To further illustrate the spatial variation of RTSIF, we show the global mean and maximum values of RTSIF in 2019 (Fig. 4). The average daily SIF has the highest values in the tropics, intermediate values in southern China, central Europe, and the eastern United States, and the lowest values in barren regions. The maximum daily SIF is found mainly in the North American corn belt, South Asia, central Europe, and tropical rainforests, consistent with the high productivity in these regions^64^. The annual average SIF and the maximum daily SIF show similar spatial patterns as those in TROPOMI SIF.

### Comparison of RTSIF with tower-based SIF

**Fig. 5:**
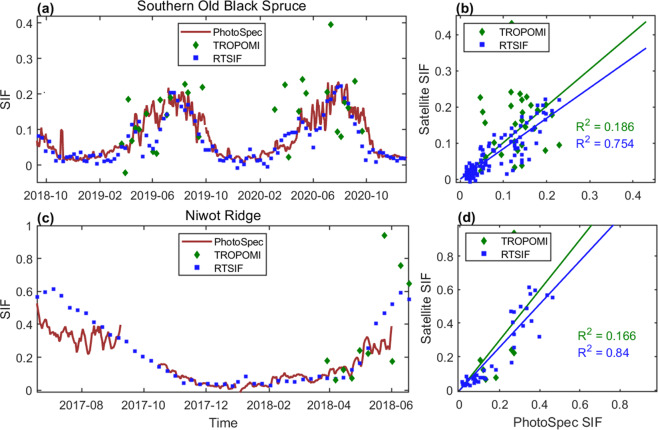
Comparison between RTSIF, TROPOMI SIF, and tower-based SIF measurements. The red line is plotted using the daily average SIF collected by PhotoSpec, presented as 5-day moving averages. The green dots represent the average of the TROPOMI SIF footprint aggregated to 8-day. The blue dot represents the RTSIF value of the pixel where the site is located. The comparison between TROPOMI SIF and RTSIF with the tower-based SIF was based on 8-day averages (**b** and **d**). All the values are in the unit of mWm^−2^nm^−1^sr^−1^.

Recently several studies have reported SIF measurements from ground towers^65–69^, providing a valuable opportunity to verify the temporal variation observed in RTSIF. We compared the tower-based SIF observations at the Southern Old Black Spruce^65^ (53.98°N, 105.12°W) and the Niwot Ridge sites^69^ (40.03°N, 105.55°W) with RTSIF. The ground tower SIF data were collected using a scanning spectrometer (PhotoSpec) for far-red (745–758 nm) SIF and retrieved by the singular value decomposition (SVD) method scaled to 750 nm. For comparison, we scaled the ground SIF to 740 nm using a wavelength scaling factor of 1.17 and aggregated the hourly data to the daily timescale^51^. Our results show good agreement between RTSIF and tower-based SIF (Fig. 5), with an R^2^ of 0.754 at the Southern Old Black Spruce site and an R^2^ of 0.84 at the Niwot Ridge site. Although mismatches were found between RTSIF and SIF measurements at the Niwot Ridge Site, which is possibly due to inconsistency between tower footprint and RTSIF pixel size and landscape heterogeneity. RTSIF captures the seasonal changes of the tower-based SIF at both sites well reprocudes, successfully locating the timing of spring onset and autumn senescence.

### Comparison of RTSIF with other SIF products

**Fig. 6:**
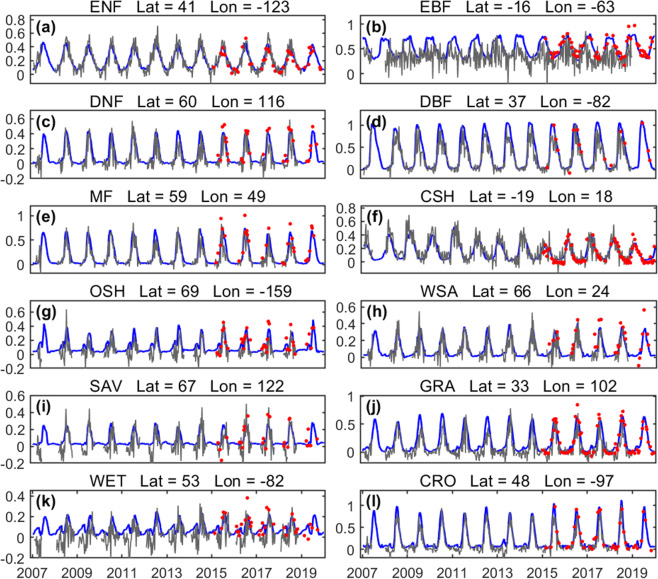
Time series of RTSIF, OCO-2 SIF, and GOME-2 SIF for selected regions. The blue line represents the RTSIF and the gray line represents the GOME-2 SIF. The red dots represent the OCO-2 SIF measurements which are not continuous. All the values are in the unit of mWm^−2^nm^−1^sr^−1^.

We further compared the RTSIF dataset with the retrievals of OCO-2 SIF and GOME-2 SIF^24,25^ (Fig. 6). OCO-2 SIF was retrieved at 757 nm, and a wavelength scale factor of 1.56 was required to convert the wavelength from OCO-2 (757 nm) to 740 nm^27^. We used OCO-2 (2015–2020) and GOME-2 (2007–2019) SIF data and aggregated all the clear-sky and good-quality measurements to 1° with an 8-day temporal resolution by using the same cloud filtering threshold (less than 0.1). All the data show similar seasonal variations in the most selected areas of typical biomes except over broad-leaf evergreen forests. The disagreement is mostly due to the low signal-to-noise ratio of GOME-2, which led the GOME-2 SIF cannot capture seasonal changes (blue lines in Fig. 6b)^50^. In addition, the large footprint of GOME-2 SIF makes it more sensitive to cloud contamination in subpixels leading to underestimated SIF values^70^. Notably, GOME-2 SIF showed large fluctuations (even negative values) during the non-growing season at some sites caused by snow contamination (Fig. 6k,i)^71,72^. RTSIF agrees well with OCO-2 SIF as the training TROPOMI SIF with high signal-to-noise ratios and spatial resolutions has demonstrated agreement with OCO-2 SIF^27^ and fills the gap where OCO-2 SIF is discontinuous both spatially and temporally.

**Fig. 7:**
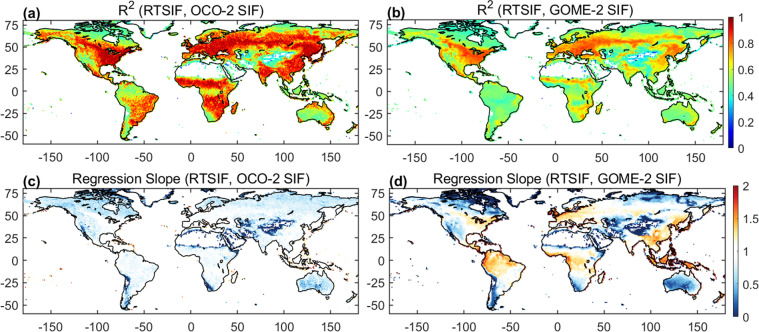
Comparison of the RTSIF, OCO-2 SIF, and GOME-2 SIF datasets. R^2^ and regression slope for RTSIF versus OCO-2 SIF (**a** and **c**) and GOME-2 SIF (**b** and **d**). The regression is forced to pass the origin. The white area represents the barren region. The data between 2015–2020 (OCO-2 SIF) and 2007–2019 (GOME-2 SIF) were used for comparison.

At the global scale, RTSIF shows good agreement with OCO-2 SIF and GOME-2 SIF in most regions with an R^2^ > 0.7 (Fig. 7a,b). The R^2^ between RTSIF and OCO-2 SIF is higher than that between RTSIF and GOME-2 SIF due to the reasons mentioned in the previous paragraph. The regression slopes of RTSIF with OCO-2 SIF and GOME-2 SIF are close to 1. However, in regions with persistent cloud cover (e.g., tropical rainforests and Western Europe), the regression slope of RTSIF with GOME-2 SIF is larger than 1 (Fig. 7d), suggesting that GOME-2 SIF is underestimated due to cloud cover in these regions. Although we filter the GOME-2 SIF data with a cloud fraction of 0.1, the large footprint size in GOME-2 SIF (~40 km) makes it impossible to remove all the subpixel cloud contamination^51^. Because our model is trained with clear-sky data (although these areas usually have high cloud coverage, there are still a large amount of clear-sky data), RTSIF is less affected by cloud cover. In addition, there is no significant increase in noise in the TROPOMI SIF due to the South Atlantic Anomaly (SSA)^73^, and RTSIF should reproduce SIF values for parts of South America with higher accuracy than OCO-2 and GOME-2 SIF. Overall it can be concluded that RTSIF provides consistent and spatially continuous SIF estimates compared to the other two products.

### Comparison of SIF with Tower GPP estimates

**Fig. 8:**
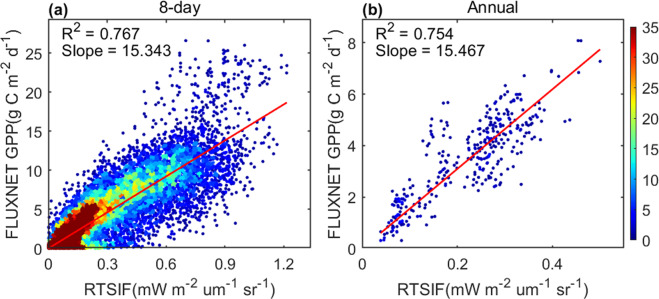
Relationship between RTSIF and FLUXNET GPP at a 8-day timescale (**a**) and the annual scale (**b**). The shading color represents the density of the scatterplot. The regression is forced to pass the origin.

To further evaluate the RTSIF product, we explored the relationship between RTSIF and GPP using GPP estimates from the FLUXNET 2015 Tier 1 dataset^74^. The daily GPP estimates were calculated using the average of GPP estimates from the nighttime (GPP_NT_VUT_REF) and daytime (GPP_DT_VUT_REF) partitioning methods^75,76^. Only the GPP estimates with more than four consecutive days of high quality (QA = 1) measurements were used when aggregated to an 8-day resolution. Considering the inconsistency between the flux tower footprint and the RTSIF pixel size, we only selected sites where the biome type in the RTSIF grid is homogeneous and the same as that at the flux tower site. We finally collected 76 sites from 171 flux sites with more than two years of GPP data. The detailed descriptions of these flux tower sites, including site code, location, and biome type are provided in Supplementary Table S3. There is a linear relationship between RTSIF and GPP in both 8-day and annual timescale (Fig. 8), indicating that RTSIF is tightly related to GPP.

To investigate whether the SIF-GPP relationship is universal for different biomes, we compared the relationship between biome-specific RTSIF and GPP (Table S4 and Fig. S2). RTSIF was in good agreement with GPP for almost all biomes at the 8-day timescale, indicating strong SIF-GPP correlations for different biomes. The agreement between RTSIF and GPP was good at the annual scale in mixed forests, woody savannas, savannas, and grasslands. GPP and RTSIF showed an overall regression slope of 15.343 (g C m^−2^  day^−1^/mWm^−2^  nm^−1^  sr^−1^) in the 8-day timescale and 15.467 (g C m^−2^ day^−1^/mWm^−2^  nm^−1^  sr^−1^) in the annual timescale, with different biomes showing significant differences. Specifically, a larger slope was found in evergreen needleleaf forests due to their distinct canopy structure, resulting in stronger reabsorption of SIF.

### Temporal patterns of the long-term RTSIF

**Fig. 9:**
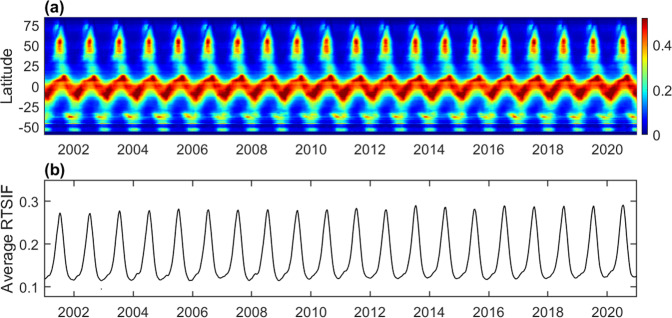
Seasonal and interannual variation of daily SIF. (**a**) Latitudinal averages of SIF for each 8-day period. (**b**) The global average of SIF for each 8-day period. All the values are in units of mWm^−2^nm^−1^sr^−1^.

We further investigated the seasonal variation of RTSIF. Fig. 9a demonstrates the seasonal variation of RTSIF in different latitudes. The northern and southern hemispheres show clear seasonal variations with repeated high values in summer. On the other hand, the tropical regions show persistently high SIF values across seasons. Globally averaged SIF shows clear seasonality (Fig. 9b).

**Fig. 10:**
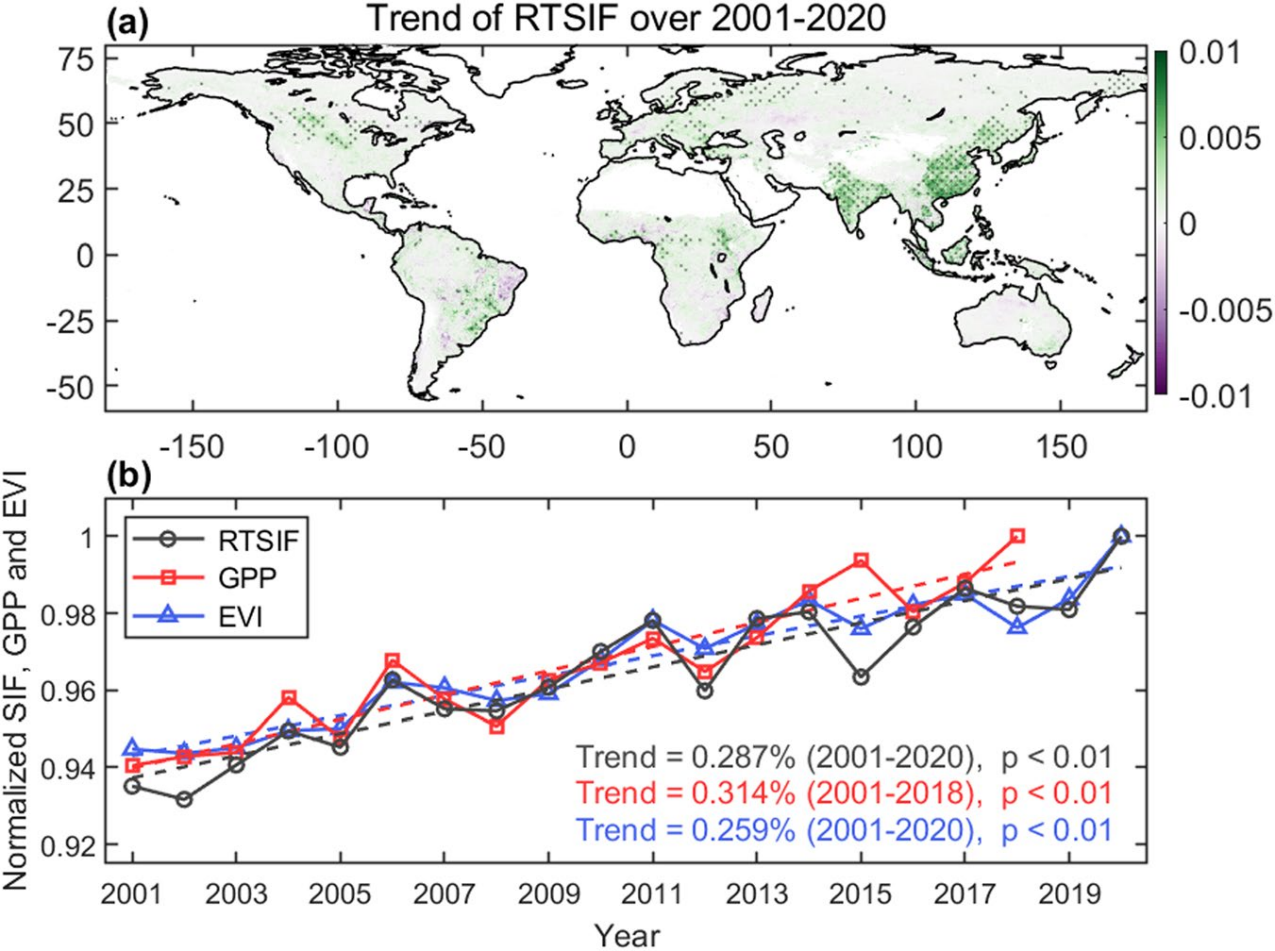
(**a**) Spatial distribution of the trends of annual average RTSIF during 2001–2020. Sen’s slope estimator is used to calculate the trend. Dots represent the locations where the trend is significant (p < 0.05) through a Mann–Kendall test. All the values are in the unit of mWm^−2^nm^−1^sr^−1^yr^−1^. (**b**) Inter-annual variations and trends of normalized global average RTSIF, EVI, and VPM GPP from 2001 to 2020.

Between 2001 and 2020, the annual average of SIF increased in China and India, and decreased in parts of the tropical rainforest (southern Amazonia and eastern Brazil), consistent with findings in previous studies^77–80^ (Fig. 10a). The global average annual RTSIF over the last 20 years has a significant positive trend (0.3% yr^−1^, p < 0.01), consistent with those observed in other reconstructed SIF products^47,50^ (Fig. S3). The interannual variability and positive trend of RTSIF are similar to those observed for MODIS EVI (enhanced vegetation index)^81^ and VPM GPP^57^, but RTSIF shows larger interannual variabilities (Fig. 10b).

## Supplementary information

Supporting Information

## Data Availability

The code for generating the RTSIF is available at https://github.com/chen-xingan/Reconstruct-TROPOMI-SIF.git.
