# Supplementary material for: A long-term reconstructed TROPOMI solar-induced fluorescence dataset using machine learning algorithms
Source: Sci Data. 2022 Jul 20;9:427. doi: 10.1038/s41597-022-01520-1 (PMC9300726; doi:10.1038/s41597-022-01520-1)
Supplement: Supplementary file 1 — Supporting Information [file 41597_2022_1520_MOESM1_ESM.pdf]

Supporting Information for "A long-term reconstructed TROPOMI solar-induced fluorescence dataset using machine learning algorithms"

Xingan Chen, Yuefei Huang, Chong Nie, Shuo Zhang, Guangqian Wang, Shiliu Chen, Zhichao Chen

Table S1. The optimized XGBoost model parameters. The most critical parameters of XGBoost include n_estimators, max_depth, min_child_weight, gamma, and learning_rate. The other hyperparameters have little or no effect on the model performance, such as subsample, silent, etc.

Parameter	Parameter Description	Value
**n_estimators**	The maximum number of boosting iterations	5000
**max_depth**	The maximum depth of a tree	10
**min_child_weight**	The minimum sum of instance weight needed in a child	6
**gamma**	The minimum loss reduction required to make a further partition on a leaf node of the tree	0.1
**learning_rate**	The step size shrinkage used in update to prevents overfitting	0.1

Table S2. Performance of XGBoost model in reproducing TROPOMI SIF for each biome types in testing data by using MCD12C1. Abbreviations are as follows: Evergreen needleleaf forests (ENF), evergreen broadleaf forests (EBF), deciduous needleleaf forests (DNF), deciduous broadleaf forests (DBF), mixed forests (MF), closed shrublands (CSH), open shrublands (OSH), woody savannas (WSA), savannas (SAV), grasslands (GRA), wetlands (WET), urban and built-up (URB), croplands (CRO), and cropland/nature vegetation mosaic(CNV).

Biome	R^2	RMSE	Slope	Number of data points
ENF	0.785	0.066	1.002	174733
EBF	0.773	0.066	1.001	101439
DNF	0.814	0.066	0.999	19285
DBF	0.946	0.072	1.000	113406

MF	0.903	0.072	1.000	304796
CSH	0.824	0.046	0.998	42562
OSH	0.749	0.054	1.002	1716157
WSA	0.875	0.066	1.001	613700
SAV	0.877	0.063	1.001	903655
GRA	0.840	0.058	1.001	2193671
WET	0.731	0.064	0.995	39856
CRO	0.930	0.074	1.000	1032526
URB	0.845	0.071	1.001	35309
CNV	0.918	0.074	1.001	36017
ALL	0.907	0.062	1.001	7327157

Table S3. FLUXNET 2015 Tier-1 sites used for evaluating RTSIF in this study.

SITE_ID	START	END	LAT	LONG	Biome
AU-Ade	2007	2009	-13.08	131.12	WSA
AU-DaP	2007	2013	-14.06	131.32	GRA
AU-Fog	2006	2008	-12.55	131.31	WET
AU-How	2001	2014	-12.49	131.15	WSA
AU-Rig	2011	2014	-36.65	145.58	GRA
AU-Stp	2008	2014	-17.15	133.35	GRA
AU-TTE	2012	2014	-22.29	133.64	GRA
AU-Ync	2012	2014	-34.99	146.29	GRA
CA-Gro	2003	2014	48.22	-82.16	MF
CA-NS1	2001	2005	55.88	-98.48	ENF
CA-NS2	2001	2005	55.91	-98.52	ENF
CA-NS3	2001	2005	55.91	-98.38	ENF
CA-NS4	2002	2005	55.91	-98.38	ENF
CA-NS5	2001	2005	55.86	-98.49	ENF
CA-NS6	2001	2005	55.92	-98.96	OSH
CA-NS7	2002	2005	56.64	-99.95	OSH
CA-Qfo	2003	2010	49.69	-74.34	ENF
CA-SF2	2001	2005	54.25	-105.88	ENF
CA-SF3	2001	2006	54.09	-106.01	OSH
CA-TP2	2002	2007	42.77	-80.46	ENF
CA-TP3	2002	2014	42.71	-80.35	ENF
CA-TP4	2002	2014	42.71	-80.36	ENF
CA-TPD	2012	2014	42.64	-80.56	DBF

CG-Tch	2006	2009	-4.29	11.66	SAV
CH-Cha	2005	2014	47.21	8.41	GRA
CH-Fru	2005	2014	47.12	8.54	GRA
CH-Lae	2004	2014	47.48	8.36	MF
CN-Cha	2003	2005	42.40	128.10	MF
CN-Cng	2007	2010	44.59	123.51	GRA
CN-Du2	2006	2008	42.05	116.28	GRA
CN-Ha2	2003	2005	37.61	101.33	WET
CN-HaM	2002	2004	37.37	101.18	GRA
CN-Qia	2003	2005	26.74	115.06	ENF
CZ-BK2	2004	2012	49.49	18.54	GRA
CZ-wet	2006	2014	49.02	14.77	WET
DE-Akm	2009	2014	53.87	13.68	WET
DE-Gri	2004	2014	50.95	13.51	GRA
DE-Lkb	2009	2013	49.10	13.30	ENF
DE-Lnf	2002	2012	51.33	10.37	DBF
DE-Obe	2008	2014	50.79	13.72	ENF
DE-RuR	2011	2014	50.62	6.30	GRA
DE-SfN	2012	2014	47.81	11.33	WET
DE-Spw	2010	2014	51.89	14.03	WET
ES-LgS	2007	2009	37.10	-2.97	OSH
FI-Let	2009	2012	60.64	23.96	ENF
FI-Lom	2007	2009	68.00	24.21	WET
FI-Sod	2001	2014	67.36	26.64	ENF
FR-Fon	2005	2014	48.48	2.78	DBF
IT-MBo	2003	2013	46.01	11.05	GRA
IT-PT1	2002	2004	45.20	9.06	DBF
IT-Tor	2008	2014	45.84	7.58	GRA
JP-MBF	2003	2005	44.39	142.32	DBF
JP-SMF	2002	2006	35.26	137.08	MF
NL-Hor	2004	2011	52.24	5.07	GRA
RU-Ha1	2002	2004	54.73	90.00	GRA
SD-Dem	2005	2009	13.28	30.48	SAV
SN-Dhr	2010	2013	15.40	-15.43	SAV
US-Goo	2002	2006	34.25	-89.87	GRA
US-IB2	2004	2011	41.84	-88.24	GRA
US-Ivo	2004	2007	68.49	-155.75	WET

US-Me2	2002	2014	44.45	-121.56	ENF
US-Me6	2010	2014	44.32	-121.61	ENF
US-Ne1	2001	2013	41.17	-96.48	CRO
US-Ne2	2001	2013	41.16	-96.47	CRO
US-Ne3	2001	2013	41.18	-96.44	CRO
US-Oho	2004	2013	41.55	-83.84	DBF
US-Prr	2010	2014	65.12	-147.49	ENF
US-SRG	2008	2014	31.79	-110.83	GRA
US-SRM	2004	2014	31.82	-110.87	WSA
US-Syv	2001	2014	46.24	-89.35	MF
US-Ton	2001	2014	38.43	-120.97	WSA
US-UMd	2007	2014	45.56	-84.70	DBF
US-Whs	2007	2014	31.74	-110.05	OSH
US-Wi3	2002	2004	46.63	-91.10	DBF
US-Wi4	2002	2005	46.74	-91.17	ENF
US-Wkg	2004	2014	31.74	-109.94	GRA

Table S4. Relationship between 8-day and annal scale from RTSIF and GPP from FLUXNET 2015 Tier 1 dataset at grid cell levels.

**Grid Cell Level GPP-SIF**	**Number of site**	**8-day**		**Yearly**	
		**R^2**	**Slope**	**R^2**	**Slope**
All	76	0.767	15.343	0.754	15.467
ENF	19	0.719	17.015	0.691	18.863
DBF	8	0.817	14.879	0.338	15.178
MF	5	0.863	14.6	0.825	15.550
OSH	5	0.622	14.903	0.589	11.699
WSA	4	0.849	16.966	0.929	17.170
SAV	3	0.819	17.127	0.996	14.806
GRA	21	0.809	14.193	0.849	14.866
WET	8	0.803	13.496	0.686	12.938
CRO	3	0.692	17.524	0.129	14.875

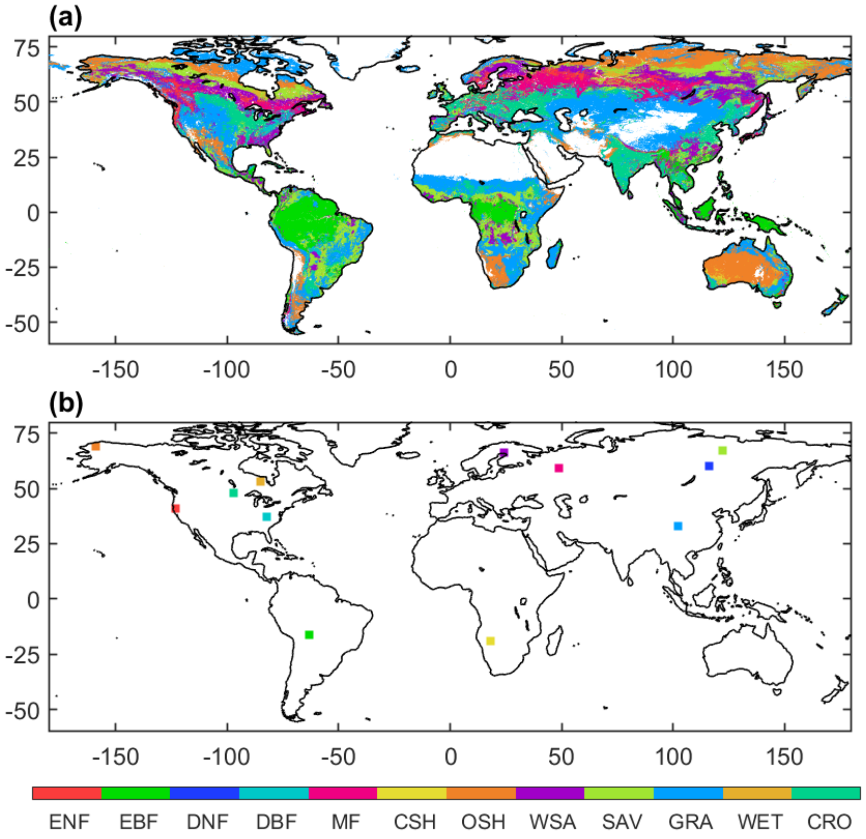

Figure S1. (a) The MODIS land cover map in 2020 from MCD12C1. (b) Location of 1° grid cells for sampling the 12 vegetated biomes.

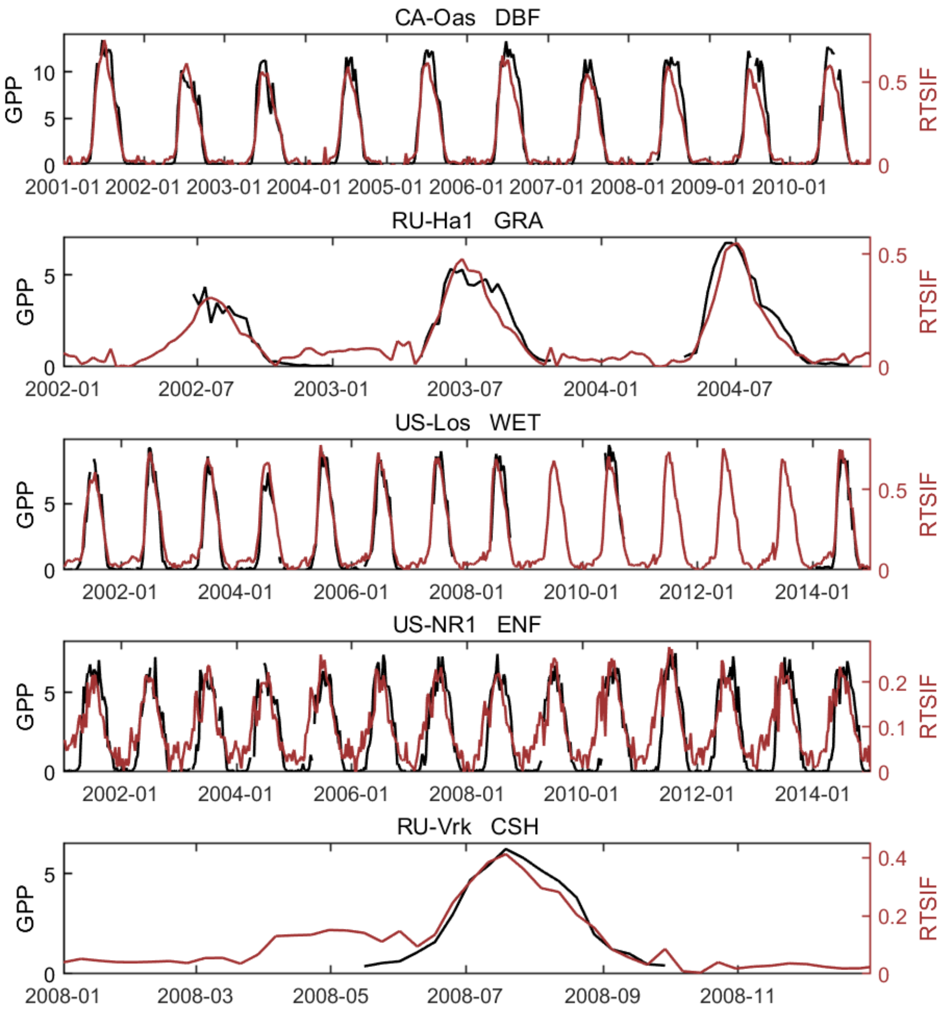

Figure S2. The seasonal cycles of RTSIF and tower-based GPP at five eddy covariance (EC) flux tower sites.

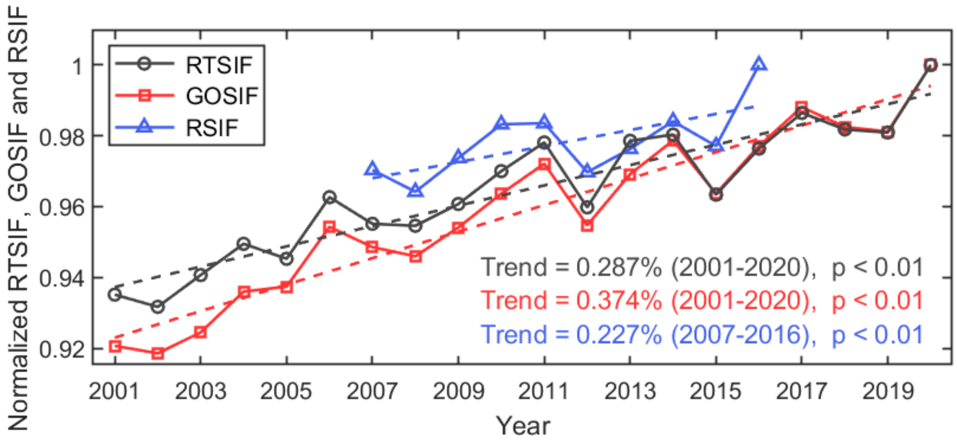

Figure S3. Inter-annual variations and trends in RTSIF, GOSIF[1], and RSIF[2] normalized by their maximum values.

Reference

1 Li, X. & Xiao, J. A Global, 0.05-Degree Product of Solar-Induced Chlorophyll Fluorescence Derived from OCO-2, MODIS, and Reanalysis Data. *Remote Sensing* **11**, 517 (2019).

2 Gentine, P. & Alemohammad, S. H. Reconstructed Solar‐Induced Fluorescence: A Machine Learning Vegetation Product Based on MODIS Surface Reflectance to Reproduce GOME‐2 Solar‐Induced Fluorescence. *Geophysical Research Letters* **45**, 3136-3146 (2018).
